# Does COVID-19 increase the long-term relapsing-remitting multiple sclerosis clinical activity? A cohort study

**DOI:** 10.1186/s12883-022-02590-9

**Published:** 2022-02-22

**Authors:** Masoud Etemadifar, Amir Parsa Abhari, Hosein Nouri, Mehri Salari, Shiva Maleki, Alireza Amin, Nahad Sedaghat

**Affiliations:** 1 Department of Neurosurgery, School of Medicine, Isfahan University of Medical Sciences, Isfahan, Iran; 2 Alzahra Research Institute, Alzahra University Hospital, Isfahan University of Medical Sciences, Isfahan, Iran; 3 Network of Immunity in Infection, Malignancy, and Autoimmunity (NIIMA), Universal Scientific, Education, and Research Network (USERN), Isfahan, Iran; 4 Functional Neurosurgery Research Center, Shohada Tajrish Comprehensive Neurosurgical Center of Excellence, Shahid Beheshti University of Medical Sciences, Tehran, Iran

**Keywords:** Demyelinating diseases, Multiple sclerosis, Disease progression, Neuroinflammation, COVID-19

## Abstract

**Background:**

Some current evidence is pointing towards an association between COVID-19 and worsening of multiple sclerosis (MS), stressing the importance of preventing COVID-19 among people with MS (pwMS). However, population-based evidence regarding the long-term post-COVID-19 course of relapsing-remitting multiple sclerosis (RRMS) was limited when this study was initiated.

**Objective:**

To detect possible changes in MS clinical disease activity after COVID-19.

**Methods:**

We conducted an observational study from July 2020 until July 2021 in the Isfahan MS clinic, comparing the trends of probable disability progression (PDP) – defined as a three-month sustained increase in expanded disability status scale (EDSS) score – and relapses before and after probable/definitive COVID-19 diagnosis in a cohort of people with RRMS (pwRRMS).

**Results:**

Ninety pwRRMS were identified with definitive COVID-19, 53 of which were included in the final analysis. The PDP rate was significantly (0.06 vs 0.19, *P* = 0.04), and the relapse rate was insignificantly (0.21 vs 0.30, *P* = 0.30) lower post-COVID-19, compared to the pre-COVID-19 period. The results were maintained after offsetting by follow-up period in the matched binary logistic model. Survival analysis did not indicate significant difference in PDP-free (Hazard Ratio [HR] [95% CI]: 0.46 [0.12, 1.73], *P* = 0.25) and relapse-free (HR [95% CI]: 0.69 [0.31, 1.53], *P* = 0.36) survivals between the pre- and post-COVID-19 periods. Sensitivity analysis resulted similar measurements, although statistical significance was not achieved.

**Conclusion:**

While subject to replication in future research settings, our results did not confirm any increase in the long-term clinical disease activity measures after COVID-19 contraction among pwRRMS.

**Supplementary Information:**

The online version contains supplementary material available at 10.1186/s12883-022-02590-9.

## Introduction

The pathomechanistic processes involved in multiple sclerosis (MS) are believed to be triggerable by systemic infections, many causing irreversible progression of the disease [1]. Naturally, this issue gains more importance during a global pandemic. Apart from its suspected neurotropism [2, 3] and similar to a wide range of other viruses [1], the severe acute respiratory syndrome coronavirus 2 (SARS-CoV-2) – etiologic cause of the current coronavirus disease 2019 (COVID-19) pandemic – could trigger exacerbation of MS by several other mechanisms, e.g., molecular mimicry, bystander activation, and epitope spreading [4]. While also considering their susceptibility to unfortunate COVID-19 outcomes [5], adopting strategies to prevent COVID-19 in the people with MS (pwMS) gains more importance, if such association could be confirmed by real-world evidence.

So far, many real-world studies have been focusing on this issue, most of which addressing MS disease activity during or shortly after COVID-19. Earlier case studies [6–9] and some population-based studies were able to confirm a positive short-term association between COVID-19 and exacerbation of relapsing-remitting MS (RRMS) [10–13]. Although this confirmation was not achieved in a study contributed by the current authors [14], the mentioned association between COVID-19 and MS disease activity could be well-explained by our current knowledge of their pathophysiology [15]. Still, the effect of COVID-19 on longer-term MS activity remains unclear.

Before the initiation of the present study, limited evidence was available regarding the clinical disease activity in people with RRMS (pwRRMS) long after recovering from COVID-19. Hence, we followed up the COVID-19-contracted pwRRMS in our clinic for a year, and investigated the possible effect of COVID-19 on their clinical disease course.

## Methods

### Settings and design

Following the strengthening the reporting of observational studies in epidemiology (STROBE) statement, we report a prospective-retrospective hybrid single-center cohort study, designed and conducted in Isfahan, Iran, from July 2020 until July 2021, aiming to address the following hypothesis: COVID-19 affects the long-term clinical activity of RRMS. In this study, we prospectively collected data pertaining to the measures of RRMS clinical activity, and compared it with retrospectively-collected data of the same participants from a year before contracting COVID-19. The hybrid design was implemented to: i) enable comparison of the RRMS clinical activity in the *same* participants before and after COVID-19, and ii) enable a more comprehensive sample recruitment. The following terms were defined and used uniformly across the manuscript:

- Pre-COVID-19 period: The one-year period ending in 2 weeks before the diagnosis of COVID-19.
- Post-COVID-19 period: The period from diagnosis of COVID-19 until the end of the study.

### Participants

The participants were recruited by screening the referrals to the Isfahan MS clinic from July until December 2020. Using the same eligibility criteria, some of the participants were recruited retrospectively by screening all of the patient records in the clinic’s registry from March until July 2020. The participants were followed up until July 2021.

Apart from providing consent to be included, the inclusion criteria were i) probable/definitive COVID-19 diagnosis made by an expert physician based the world health organization (WHO) definitions [16], and ii) definitive RRMS diagnosis made by a neurologist according to the 2017 McDonald criteria [17] at least 54 weeks before COVID-19 diagnosis. The exclusion criteria were i) absence for the routine three-month follow-up visit and ii) Diagnosis of secondary progressive MS (SPMS). Screening for COVID-19 was based on the reports of the pwRRMS themselves, proved with documentations of highly-suggestive symptoms, lung computer tomography (CT), and/or reverse transcriptase polymerase chain reaction (RT-PCR) either provided by the participants from other reliable sources (i.e. other laboratories, clinics, hospitals) or from our own clinic registry. Cases with suspected COVID-19 based on the WHO definitions [16] were not considered eligible for the study. No specific sample size was pre-defined for this study.

### Variables and measurements

After confirmation of eligibility, the following measures were collected for each participant: age, sex, comorbidity status, disease duration, disease-modifying therapy (DMT), COVID-19 diagnosis method, onset, and severity. In order to compensate for the relatively-small study scale in size and duration and to make it more sensitive to the possible post-COVID-19 increase in RRMS clinical activity, we used *probable* disability progression (PDP) as the primary endpoint, defined as a rise in expanded disability status scale (EDSS) score, sustained for at least 3 months. Acute relapse of MS was considered the secondary outcome, defined as the development of new or worsening of pre-existing neurological symptoms lasting at least 24 h, preceded by a 30-day stable or improving neurological state, and in the absence of fever, infection or steroid withdrawal. Participants were frequently visited by their neurologist and underwent thorough neurological examinations, enabling us to collect outcome data prospectively from each individual upon each visit. As a point of comparison, we used the clinic registry to retrieve the outcome data pertaining to the pre-COVID-19 period of each participant, after confirmation of eligibility. Hence, all data pertaining to the pre-COVID-19 disease status of the participants was collected retrospectively. Some post-COVID-19 data pertaining to the participants who were included based on their records in the clinic registry was also collected retrospectively similar to the pre-COVID-19 data.

### Statistical analysis

Cumulative outcome measures from the ending timepoints of pre- and post-COVID-19 periods were compared using Wilcoxon signed-rank and McNemar tests. A binary logistic model was used to compare RRMS clinical activity between the people who were hospitalized due to COVID-19 and the ones who were not. As the Wilcoxon signed-rank test does not account for the variable post-COVID-19 follow-up periods, a matched binary logistic model offset by the follow-up durations was used to compare the outcome measures i.e. the cumulative PDP and relapse rates at the ending timepoints of the pre- and post-COVID-19 periods. Kaplan-Meier survival plots and cox regression models were used to compare the PDP-free and relapse-free survivals *during* the pre- and post-COVID-19 periods. Sensitivity analysis included repeating all analyses in the subgroup of prospectively recruited participants. Multivariable analysis was not applicable, as the comparisons were being made between *the same* participants before and after a certain timepoint, who were regarded as two dependent samples in the analysis. Results with *P* values below 0.05 were referred to as significant; however, caution is advised with interpreting the results of individual statistical tests as significant. To reduce possible bias, and for privacy protection of the participants, all data were anonymized before analyses, for which IBM SPSS v.23 for macOS was used.

## Results

### Participants

**Fig. 1:**
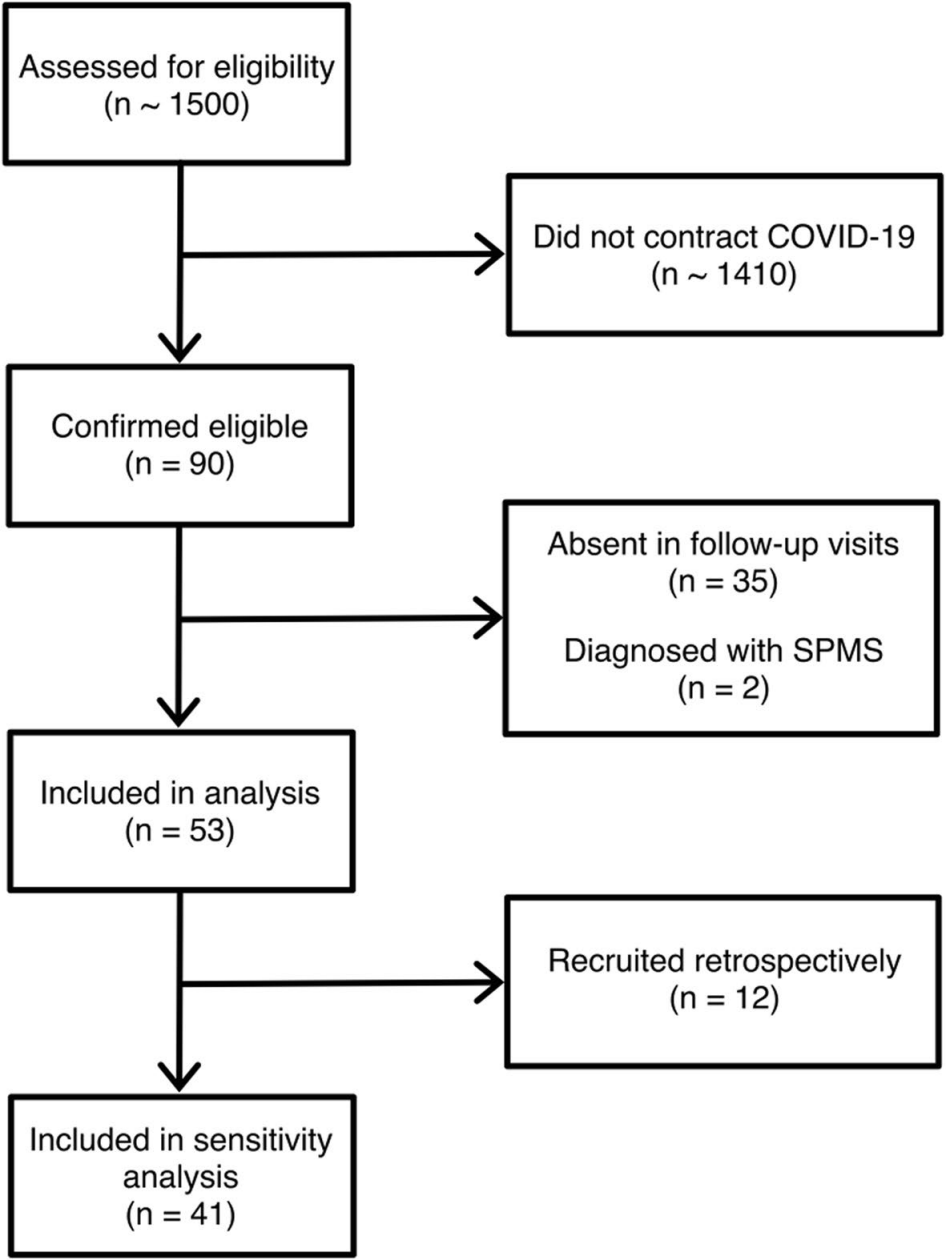
Study flow diagram

The detailed flow diagram of the study can be interpreted from Fig. 1. Thirty seven pwRRMS were excluded after eligibility confirmation, two were diagnosed with SPMS, and the other 35 opted for remote visits and were therefore, absent in the follow-up visits. As precise and objective EDSS assessment was not possible in tele-visits, while also considering the over-sensitive primary endpoint of the study, the remotely-visited pwMS were not included in the analyses. A total of 53 pwMS completed the study and were included in the analyses, of which 41/53 were recruited prospectively, and 12/53 were recruited based on their records in the clinic registry (Fig. 1). It should be pointed that a portion of the participants may have been the same as a previously-conducted study contributed by some authors of the present study [14] as the same center was used for identification and enrollment of participants, despite the two studies being conducted separately and independently.

### Descriptive data and analysis results

The descriptive baseline and outcome data pertaining to the participants can be interpreted from Table 1. No change of DMT was recorded for the pwMS during the study period, and none of the EDSS assessments were carried out during disease flares. 6/53 of the pwMS were hospitalized due to COVID-19 but managed to recover without complications like the others. 4/6 hospitalized pwMS were on dimethyl fumarate, 1/6 was on rituximab, and 1/6 was on interferon-beta. Clinical activity of RRMS was increased in the pwRRMS who were hospitalized due to COVID-19 (PDP: Odds Ratio [OR] [95% Confidence Interval (CI)]: 4.50 [0.34, 58.92], *P* = 0.25; relapses: OR [95% CI]: 2.11 [0.33, 13.38], *P* = 0.43), suggesting a possible positive correlation between severity of COVID-19 and the subsequent RRMS clinical activity, however, this could not be confirmed due to lack of statistical significance.

**Table 1:** Descriptive data of participants

**Variable**	**Final participants (*n*** **= 53)**		
Mean age (SD) [years]	38.42 (8.77)		
Sex (female:male)	45:8		
Comorbidity (%)	None: 43 (81.13) HTN/CV: 3 (5.66) Hypothyroidism: 2 (3.77) DM: 4 (7.55)		
Median MS duration (IQR) [years]	10 (13)		
DMT (%)	No DMT: 3 (5.66) IFNs: 4 (7.54) DMF: 21 (39.62) TFN: 1 (1.89) GA: 1 (1.89) FNG: 12 (22.64) RTX: 9 (16.98) AZA: 2 (3.77)		
COVID-19 diagnosis type (%)	RT-PCR: 30 (56.60) CT: 14 (26.42) Both: 9 (16.98)		
COVID-19 severity (%)	No hospitalization: 47 (88.68) Hospitalization: 6 (11.32)		
Mean post-COVID-19 follow-up (SD) [months]	10.58 (2.48)		
***Outcome***	**Pre-COVID-19 period endpoint**	**Post-COVID-19 period endpoint**	***P*** **value**
Median EDSS (IQR)	1.5 (1)	1.5 (1)	0.08*
Number of patients with probable disability progression (%)	10 (18.87)	3 (5.66)	0.04**
Number of patients experiencing relapses (%)	16 (30.19)	11 (20.75)	0.30**

*Wilcoxon signed-rank test

**McNemar test

*Abbreviations*: *SD* standard deviation, *HTN* hypertension, *CV* cardiovascular, *DM* diabetes mellitus, *MS* multiple sclerosis, *IQR* interquartile range, *DMT* disease-modifying therapy, *IFN* interferon, *DMF* dimethyl fumarate, *TFN* teriflunomide, *GA* glatiramer acetate, *FNG* fingolimod, *RTX* rituximab, *AZA* azathioprine, *RT-PCR* reverse transcription polymerase chain reaction, *CT* computed tomography, *EDSS* expanded disability status scale;

Furthermore, the PDP rate was significantly (0.06 vs 0.19, *P* = 0.04), and the relapse rate was insignificantly (0.21 vs 0.30, *P* = 0.30) lower post-COVID-19, compared to the pre-COVID-19 period. The results were mainained after offsetting by follow-up period in the matched binary logistic model (PDP: OR [95% CI]: 0.29 [0.09, 0.95], *P* = 0.04; relapse: OR [95% CI]: 0.69 [0.32, 1.51], *P* = 0.36). One-tailed hypothesis testing indicated that COVID-19 is unlikely to increase the long-term PDP rate in pwRRMS (one-tailed *P* = 0.02). This confirmation was not achieved regarding relapse rates (one-tailed *P* = 0.18).

**Fig. 2:**
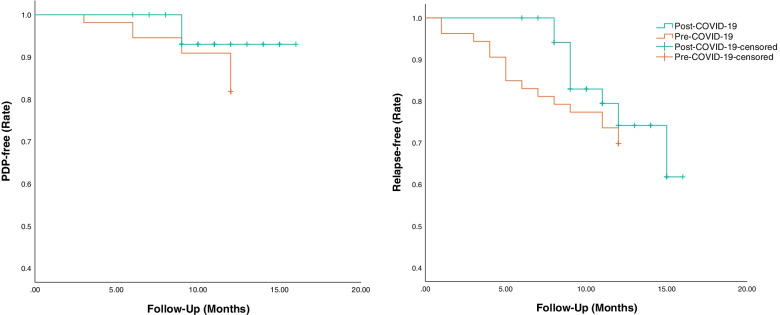
Kaplan-Meier survival plots

Furthermore, the Kaplan-Meier plots (Fig. 2) and cox regression analyses did not confirm any significant difference in the PDP-free (Hazard Ratio [HR] [95% CI]: 0.46 [0.12, 1.73], *P* = 0.25) and relapse-free (HR [95% CI]: 0.69 [0.31, 1.53], *P* = 0.36) survivlas between the pre- and post-COVID-19 periods. Still, according to one-tailed hypothesis testing, the possible post-COVID-19 decrease in both PDP-free (one-tailed *P* = 0.12) and relapse-free (one-tailed *P* = 0.18) survivals could not be ruled out on the basis of these results.

The sensitivity analysis among the 41/53 prospectively-recruited participants showed similar measurements, although none of the results achieved statistical significance, possibly due to the limited subgroup size (Supplementary Material).

## Discussion

The present study results did not show any increase in PDP and relapse rates in pwRRMS after COVID-19 contraction. A decrease was observed in the cumulative PDP rate of pwRRMS after recovering from COVID-19, which may be explained by a decoy effect of COVID-19, diverting the inflammatory cells to the lungs from the central nervous system. This decrease did not reach statistical significance in the sensitivity analysis, and remains to be confirmed in replicative studies, however, we confirmed that the cumulative PDP rates are, at least, unlikely to be *increased* long after recovering from COVID-19. Moreover, the study lacked enough statistical power to rule out the possible increase in cumulative relapse rates, and the possible decrease in PDP-free and relapse-free survivals after recovering from COVID-19. Other limitations of this study include the relatively small-scale and observational nature, possible ascertainment/measurement bias, inappropriate identification of confounding factors, high female-to-male ratio suggesting possible sampling bias, and possible missing of asymptomatic/mild COVID-19 cases. We tried to encounter the small scale by utilizing a modified primary endpoint, i.e., *probable* disability progression instead of *confirmed* disability progression. This modification made the study more sensitive, although it may have introduced false-positive outcome measurements. Despite using this over-sensitive endpoint, most of the comparisons did not show any statistically-significant difference between the two periods of pre- and post-COVID-19 contraction, hence, the utilization of the standard endpoint i.e. *confirmed* disability progression, which is less sensitive but more specific, is doubted to have ceaused any significant shift in the conclusions. Furthermore, the male population of our study was limited, limiting its generalisibility to the male pwRRMS, as their overall courses of COVID-19 and RRMS differs from the female pwRRMS. Altogether, the findings of this study are subject to replication in future settings and should be taken into consideration with caution.

It has been hypothesized that coronaviruses can exacerbate/induce MS via different mechanisms, e.g., direct invasion, providing activation signals for autoreactive lymphocytes, and disrupting the blood-brain barrier by inducing the release of pro-inflammatory cytokines [18, 19]. Regarding SARS-CoV-2, earlier case studies [6–9] and some population-based evidence [10–13] backed these hypotheses, at least in short-term. However, the present study and another study in the same setting [14] were not able to confirm any longer-term relationship between COVID-19 and clinical MS activity. These discrepencies may be explained by heterogenous definitions of an MS relapse, e.g., Garjani et al. did not include prior 30-day stable state, lasting at least 24 h, and most important of all, the absence of fever, infection or steroid withdrawal, in their definition of an MS exacerbation [13]. Also, the probability of confirmation, ascertainment, and/or selection biases should be acknowledged, e.g., a considerable ascertainment/confirmation and selection biases were present in the study by Barzegar et al. [10], as explained in a later commentary by Sedaghat [11]. Nevertheless, the reports and theories in support of SARS-CoV-2 being able to facilitate MS progression cannot be disregarded until larger studies with more statistical power could rule them out. The rarity of neuro-invasive SARS-CoV-2 infection and the small and observational nature of the conducted studies could explain the inconsistencies, in which case, more extensive and reliable studies in the future are warranted to provide theory-matched evidence. Otherwise, the missing piece of the puzzle in the immunopathology of COVID-19, which prevents the mentioned mechanisms from triggering MS exacerbation/progression, remains to be investigated.

## Conclusion

Unlike the previous studies in other settings, our preliminary cohort study failed to detect any increase in measures of clinical disease activity in pwRRMS. Experts are advised to replicate such studies with larger sample sizes in order to enable provision of evidence-based individualized care for the pwRRMS.

## Supplementary Information

**Additional file 1.** Supplementary Material.

## Data Availability

The datasets generated and/or analysed during the current study are not publicly available to ensure privacy protection of the participants, but are available from the corresponding author on reasonable request.

## Abbreviations

MS: Multiple sclerosis
SARS-CoV-2: Severe acute respiratory syndrome coronavirus 2
COVID-19: Coronavirus disease 2019
pwMS: People with multiple sclerosis
RRMS: Relapsing-remitting multiple sclerosis
pwRRMS: People with relapsing-remitting multiple sclerosis
STROBE: Strengthening the reporting of observational studies in epidemiology
WHO: World health organization
SPMS: Secondary progressive multiple sclerosis
CT: Computer tomography
RT-PCR: Reverse transcriptase polymerase chain reaction
DMT: Disease-modifying therapy
PDP: Probable disability progression
EDSS: Expanded disability status scale
OR: Odds ratio
CI: Confidence interval
HR: Hazard ratio
