# Supplementary material for: Does COVID-19 increase the long-term relapsing-remitting multiple sclerosis clinical activity? A cohort study
Source: BMC Neurol. 2022 Feb 22;22:64. doi: 10.1186/s12883-022-02590-9 (PMC8861623; doi:10.1186/s12883-022-02590-9)
Supplement: Supplementary file 1 — Additional file 1. Supplementary Material. [file 12883_2022_2590_MOESM1_ESM.docx]

# Supplementary Material

## Results of Sensitivity Analysis

Among the 41/53 prospectively-recruited participants (sensitivity analysis), McNemar test showed no significant difference in PDP rates (0.07 vs 0.19, P = 0.12) and relapse rates (0.24 vs 0.19, P = 0.75) between the ending timepoints of pre- and post-COVID-19 periods. The matched binary logistic model offset by follow-up period showed no significant difference in odds of PDP (OR [95% confidence interval]: 0.41 [0.13, 1.34], P = 0.14) and odds of relapse (OR [95% confidence interval]: 0.99 [0.45, 2.17], P = 0.99) between the endpoints of pre- and post-COVID-19 periods, and the Kaplan-Meier survival plot and cox regression did not indicate significant difference of PDP hazard (HR [95% confidence interval]: 0.78 [0.19, 3.26], P = 0.78) and relapse hazard (HR [95% confidence interval]: 1.05 [0.44, 2.52], P = 0.91) during the pre- and post-COVID-19 periods.
